# Indoxyl Sulfate in the Gut–Kidney Axis: Pathophysiology and Clinical Significance in CKD-Associated Colorectal Cancer

**DOI:** 10.3390/toxins18020072

**Published:** 2026-01-30

**Authors:** Hidehisa Shimizu, Toshimitsu Niwa

**Affiliations:** 1 Faculty of Life and Environmental Sciences, Shimane University, 1060 Nishikawatsu-cho, Matsue 690-8504, Shimane, Japan; 2 Graduate School of Natural Science and Technology, Shimane University, 1060 Nishikawatsu-cho, Matsue 690-8504, Shimane, Japan; 3 The United Graduate School of Agricultural Sciences, Tottori University, 4-101 Koyama-Minami, Tottori 680-8553, Tottori, Japan; 4 Estuary Research Center, Shimane University, 1060 Nishikawatsu-cho, Matsue 690-8504, Shimane, Japan; 5 Interdisciplinary Center for Science Research, Shimane University, 1060 Nishikawatsu-cho, Matsue 690-8504, Shimane, Japan; 6 Institute of Agricultural and Life Sciences, Academic Assembly, Shimane University, 1060 Nishikawatsu-cho, Matsue 690-8504, Shimane, Japan; 7 Faculty of Medical Sciences, Shubun University, 6 Nikko-cho, Ichinomiya 491-0938, Aichi, Japan

**Keywords:** Chronic Kidney Disease, colorectal cancer, indoxyl sulfate, gut–kidney axis, *Fusobacterium nucleatum*, tumor microenvironment, uremic toxins, Liquid Biopsy, Aryl Hydrocarbon Receptor, Tryptophan Metabolism

## Abstract

Chronic Kidney Disease (CKD) and Colorectal Cancer (CRC) share a profound epidemiological link, supported by Mendelian randomization studies suggesting causality. This review articulates a refined Gut–Kidney Axis, focusing on the pathophysiology of indole-derived uremic toxins. CKD-induced dysbiosis drives hepatic synthesis and systemic accumulation of indoxyl sulfate, which is proposed to promote carcinogenesis via Aryl Hydrocarbon Receptor (AhR) and Akt signaling, ultimately upregulating *c-Myc* and *EGFR*. We propose a two-compartment model: while systemic indoxyl sulfate reflects the total gut indole pool (mainly from planktonic bacteria), adherent bacteria like *Fusobacterium nucleatum* may create high-concentration indole hotspots within the tumor microenvironment. Clinically, we advocate for protein-independent DNA methylation biomarkers (SEPT9, SDC2) to avoid renal confounding. Furthermore, we propose a novel diagnostic panel integrating serum indoxyl sulfate (systemic load) and urinary indoxyl sulfate (gut production) to guide therapy. Therapeutically, targeting upstream drivers (AhR/Akt) may bypass resistance to anti-EGFR therapies in *KRAS*-mutated tumors. We also discuss the repurposing of the oral adsorbent AST-120 and emerging bacteriophage therapies as strategies to disrupt this oncogenic axis. This review offers a comprehensive framework for stratified management of CKD-associated CRC.

## 1. Introduction

Chronic Kidney Disease (CKD) and Colorectal Cancer (CRC) are two globally significant health burdens, both of which are increasing in prevalence. Although they have traditionally been managed as separate clinical entities, a growing body of evidence suggests a strong and potentially causal link between them. This review aims to synthesize the current understanding of this relationship. To provide a comprehensive overview, we structure this review to progress from the epidemiological association to the underlying molecular mechanisms, and finally to the implications for clinical practice and future research. We begin by establishing robust epidemiological evidence for an association between CKD and CRC, highlighting high-risk subgroups. We then delve into the central pathophysiological hypothesis, exploring the complex Gut–Kidney Axis. Following this mechanistic exploration, we address the significant clinical challenges in diagnosis and biomarkers. Finally, we evaluate therapeutic strategies and outline recommendations for personalized interventions. While previous reviews have touched upon aspects of the gut–kidney axis, this review uniquely integrates epidemiological evidence with a refined ‘two-compartment’ pathophysiological model of indole-derived uremic toxins, bridging the gap between systemic and local effects to propose a comprehensive framework for CKD-associated CRC.

It is crucial to acknowledge that the uremic milieu is complex and comprises various retention solutes. Alongside indoxyl sulfate, other protein-bound uremic toxins such as p-cresyl sulfate and small water-soluble solutes like trimethylamine *N*-oxide (TMAO) play significant roles in CKD-associated pathology. p-cresyl sulfate has been strongly linked to cardiovascular mortality and systemic oxidative stress [1,2], while TMAO, derived from gut microbial metabolism of choline, is implicated in atherosclerosis and potentially colorectal carcinogenesis via inflammatory pathways [3,4,5,6]. Although these metabolites likely act in concert to promote a pro-tumorigenic environment, this review specifically focuses on indoxyl sulfate. This distinction is made because indoxyl sulfate possesses a unique and potent ability to act as an endogenous ligand for the Aryl Hydrocarbon Receptor (AhR), thereby directly driving oncogenic transcription factors like c-Myc, a mechanism distinct from the general oxidative stress induced by other toxins.

Within this framework, the unique behavior of specific gut microbes, such as *Fusobacterium nucleatum* (*F. nucleatum*), adds another layer of complexity. Its unique ability to firmly adhere to tumor cells potentially creates a distinct microenvironment that amplifies pro-carcinogenic signals through local indole production, making the interplay between local microbial behavior and systemic toxins a particularly compelling area of investigation. Recently, the potential of this “local-systemic” intersection was highlighted in a conceptual framework [7]. This framework posits that while systemic indoxyl sulfate is driven by the total gut indole pool, local carcinogenesis may be accelerated by adherent bacteria forming high-concentration indole “hotspots” directly on the tumor surface.

## 2. The Intersecting Burden of Chronic Kidney Disease and Colorectal Cancer: An Epidemiological Certainty

This section provides foundational evidence for the link between CKD and CRC. It progresses the argument from statistical association to evidence supporting causality, identifies high-risk populations, and details the profound negative impact of this comorbidity on the clinical outcomes.

### 2.1. Quantifying the Risk: Evidence Synthesis from Meta-Analyses

Multiple large-scale meta-analyses have consistently demonstrated a statistically significant increase in CRC risk among patients with CKD [8,9] (Table 1). To fully appreciate the robustness of this association, it is important to distinguish between the different types of relative risk indicators reported in the literature. Studies typically utilize either the Standardized Incidence Ratio (SIR), which compares observed cases in patients to expected cases in the general population, or the Risk Ratio (RR) and Hazard Ratio (HR), which compare risks between exposed and unexposed groups within controlled cohorts. A seminal meta-analysis of 14 studies reported a pooled SIR for CRC of 1.33 (95% CI, 1.30–1.36), indicating a 33% increased relative incidence compared with the general population [8]. Corroborating this, a more recent comprehensive analysis encompassing eight cohort studies with over 1.3 million participants reported a nearly identical pooled RR of 1.332 (95% CI, 1.084–1.637) [9]. The remarkable consistency of these values—derived from different statistical methodologies and populations—underscores the validity of the association. This elevated risk appears to be a fundamental feature of renal dysfunction, persisting even after kidney transplantation [10]. Furthermore, evidence indicates that the risk is not static but increases with the severity of the disease. Specifically, a nationwide cohort study demonstrated that individuals with a reduced estimated Glomerular Filtration Rate (eGFR) had a significantly higher risk of adverse outcomes, strongly suggesting a dose–response relationship between CKD severity and systemic tumorigenic risks [11]. These findings represent more than a mere statistical signal; they indicate a substantial public health burden. Given the high global prevalence of CKD [12], a roughly 33% increase in relative risk (potentially higher in advanced stages) translates into a considerable number of excess CRC cases attributable to renal dysfunction. This establishes a compelling imperative for enhanced surveillance in this population.

### 2.2. From Correlation to Causation: Evidence Supporting a Causal Link via Mendelian Randomization

While meta-analyses suggest a strong association, traditional observational studies face a fundamental limitation: CKD and CRC share numerous potent confounding factors—such as obesity, diabetes mellitus, poor diet, and smoking—that are extremely difficult to disentangle statistically. To overcome this, recent research has turned to Mendelian Randomization (MR), which offers more compelling evidence of causality [9]. MR leverages genetic variants associated with CKD susceptibility as “instrumental variables.” Because these genetic variants are randomly allocated at conception and precede lifestyle choices, MR functions similarly to a randomized controlled trial provided by nature, effectively minimizing environmental confounders. A pivotal study employing this approach reported that genetically predicted CKD was significantly associated with an increased risk of CRC (Odds Ratio [OR] 1.171, 95% CI 1.063–1.289) [9]. This MR finding moves the discussion beyond mere statistical association. It suggests that the pathophysiological state of CKD itself—potentially through mechanisms such as the systemic accumulation of uremic toxins and chronic inflammation explored later in this review—actively promotes colorectal carcinogenesis, rather than the association being solely due to shared risk factors [12]. Without the support of these MR findings, the pursuit of specific biological mechanisms, such as the role of indoxyl sulfate, would remain far more speculative. However, it is crucial to interpret these findings with appropriate scientific caution. We must acknowledge inherent methodological limitations, such as potential “weak instrument bias” or horizontal pleiotropy. Specifically, horizontal pleiotropy remains a concern, where genetic variants associated with CKD might independently influence CRC risk through pathways unrelated to kidney function (e.g., via shared metabolic or inflammatory traits). Nevertheless, the available MR evidence provides the strongest support to date for the hypothesis that CKD is not merely a bystander but a direct contributing factor to colorectal carcinogenesis.

### 2.3. High-Risk Populations: Delineating the Disproportionate Burden and Synergistic Risks

The causal relationship between CKD and CRC does not appear to be uniform across all populations. Subgroup analyses have revealed a markedly higher risk in specific demographics, driven by distinct pathophysiological and metabolic mechanisms:

- Younger Patients (<50 years): Paradoxically, the relative risk seems most pronounced in the young. The MR analysis showed an RR of 2.119 (a 112% increase) in this demographic [9]. This is corroborated by a population-based cohort study that reported an even higher Hazard Ratio (HR) of 3.7 (a 270% increase) in this age group [13]. This disproportionate risk may be explained by the concept of “accelerated aging” or “inflammaging” associated with CKD [14,15,16,17]. Specifically, uremic toxins such as indoxyl sulfate have been shown to downregulate Klotho [18]. Importantly, Klotho functions as a physiological antagonist of the Wnt/β-catenin pathway. Its depletion by indoxyl sulfate may lead to the “disinhibition” of Wnt signaling, thereby creating a permissive environment for carcinogenesis that synergizes with the direct activation of this pathway discussed in Section 3. Furthermore, recent evidence suggests that indoxyl sulfate induces ‘trained immunity’ in monocytes via an AhR-dependent arachidonic acid pathway. This epigenetic reprogramming of immune cells perpetuates a state of chronic low-grade inflammation, providing a mechanistic basis for the persistent ‘inflammaging’ observed in CKD patients [19]. Consequently, CKD creates a pro-carcinogenic systemic milieu in younger individuals that mimics the biological phenotype typically seen in the elderly.
- Women: The association appears stronger in women, with an RR of 1.550 (a 55% increase) [9]. The biological basis for this sex-specific risk remains under investigation but likely involves hormonal factors. Estrogen is generally considered protective against CRC development, potentially via Estrogen Receptor β (ERβ) signaling [20,21,22,23]. In patients with CKD, hormonal dysregulation and hypothalamic–pituitary–gonadal (HPG) axis suppression are common [24,25,26,27,28]. We hypothesize that the disruption of this protective estrogenic shield in female CKD patients may render them more susceptible to the pro-oncogenic effects of uremic toxins, although this requires specific validation.
- The Synergistic Effect of Comorbidities: A powerful synergistic effect exists where women with both CKD and diabetes mellitus (DM) have a significantly higher risk (HR 2.00 vs. healthy controls) compared to those with either condition alone [29]. This finding suggests that the metabolic dysregulation inherent in diabetes amplifies the uremic risk, creating a super-high-risk phenotype that warrants aggressive surveillance.
- Data Stratification and Limitations: It is important to note a limitation in current epidemiological evidence: most large-scale meta-analyses do not provide granular risk stratification by narrow age bands (e.g., decadal breakdown) or detailed CKD stages (e.g., G3a vs. G3b). As highlighted in our response to the reviewers, future prospective cohorts are strictly necessary to define absolute risk estimates more precisely across these specific subgroups to guide personalized screening guidelines.

These findings suggest the need for a stratified approach to clinical surveillance. The exceptionally high risk in younger CKD patients is particularly alarming as it intersects with the globally rising trend of early-onset CRC (EOCRC) [30]. This suggests that CKD acts as a potent risk amplifier, accelerating carcinogenesis in populations already facing an increasing cancer burden.

### 2.4. Clinical Ramifications: The Detrimental Impact of CKD on Postoperative Outcomes and Long-Term Survival

The presence of CKD significantly worsens the prognosis of patients with CRC, acting as a major determinant of both short-term surgical outcomes and long-term survival. While the overall rates of minor postoperative complications may be comparable to non-CKD patients, the physiological toll on CKD patients is distinct and severe. Patients with CKD face a higher risk of cardiovascular morbidity and more than double the 30-day mortality rate following CRC resection (4.8% vs. 2.1%) [30]. Comorbid renal dysfunction is broadly associated with inferior outcomes, including a higher incidence of in-hospital mortality and infection [31]. Furthermore, studies have indicated that CKD is highly prevalent in patients undergoing CRC surgery, suggesting that renal dysfunction serves as a critical proxy for increased perioperative risk and systemic frailty [32]. Regarding long-term outcomes, Kaplan–Meier analyses have consistently demonstrated poorer Overall Survival (OS) and Disease-Free Survival (DFS) in the CKD population compared to patients with normal renal function [30]. However, discerning the cause of this mortality is critical for clinical management. A pivotal multivariate analysis by Currie et al. identified CKD as an independent prognostic factor specifically for non-cancer death (HR = 1.82) but not for cancer-specific mortality [33]. This distinction suggests that the poorer prognosis in this population is driven primarily by CKD-related complications—such as cardiovascular events and uremic toxicity—rather than by a more aggressive cancer biology per se. This “non-cancer death” finding reveals that the primary therapeutic challenge in CKD-CRC patients extends beyond the tumor itself to the rigorous management of systemic frailty and competing risks.

## 3. Indoxyl Sulfate and Beyond: Molecular Linchpins in the Gut–Kidney Axis

This section dissects the central mechanistic hypothesis, focusing on indoxyl sulfate and *F. nucleatum*-mediated local effects. This demonstrates that these factors are not merely passive toxins but products and mediators of a vicious cycle driven by CKD itself.

### 3.1. CKD-Induced Dysbiosis and the Gut–Kidney Axis: Fueling the Toxin Factory

The link between the gut, kidneys, and colon is bidirectional and synergistic [34]. The pathophysiology begins with CKD. As kidney function declines, uremic toxins, particularly urea, accumulate. This urea is excreted into the gut lumen, where it is metabolized by bacterial urease to ammonia, altering the intestinal pH and environment [35,36,37,38]. This process, known as gut dysbiosis, creates selective pressure within the uremia-associated intestinal milieu that favors the proliferation of specific pathogenic and putrefactive bacteria, including indole-producing species such as *F. nucleatum* [7,35]. This uremic environment compromises the integrity of the intestinal epithelial barrier, leading to a “leaky gut” condition that permits the translocation of bacterial products (such as lipopolysaccharide) and fuels systemic inflammation [39,40].

### 3.2. The Pathophysiological Pathway: Systemic Indoxyl Sulfate and Local Indole—A Refined Model

Indoxyl sulfate, a protein-bound uremic toxin [1], originates from dietary tryptophan intake. In the dysbiotic gut environment described above, indole-producing bacteria metabolize tryptophan into indole via enzymes such as tryptophanase. This indole is absorbed into the portal circulation and hydroxylated in the liver to form indoxyl sulfate, which then circulates systemically [34]. While the liver-specific OATP1B1 facilitates systemic clearance, colorectal cancer cells, such as HCT-116, frequently overexpress the cancer-specific variant Ct-OATP1B3. Unlike the liver-type OATP1B3 (Lt-OATP1B3) which localizes to the plasma membrane, recent studies indicate that Ct-OATP1B3 primarily localizes to the cytoplasm (lysosomes) [41]. This suggests Ct-OATP1B3 itself may not mediate the initial uptake of indoxyl sulfate. Upon intracellular accumulation, indoxyl sulfate activates pro-oncogenic signals. Importantly, we have previously identified that organic anion transporters OAT1 and OAT3 are critically involved in the tubular transport and renal clearance of indoxyl sulfate, and their dysfunction in CKD directly leads to indoxyl sulfate accumulation [42].

As a point of scientific rigor, it is important to acknowledge the quantitative dominance of *E. coli* in the gut. Due to its high abundance and tryptophanase activity, *E. coli* is likely the primary contributor to the total systemic indole pool [43]. However, *E. coli* is typically a planktonic (free-floating) organism in the gut lumen. Indole produced by such planktonic bacteria is rapidly diluted in the luminal contents before being absorbed, primarily contributing to systemic circulating levels (i.e., serum indoxyl sulfate) rather than creating a high local concentration at a specific tissue site. In contrast, while not all strains of *F. nucleatum* possess the *tnaA* (tryptophanase) gene required for indole synthesis, many clinical isolates from CRC patients demonstrate this capability [7,44]. Crucially, the specific pathogenicity of *F. nucleatum* likely stems from its “location” and “lifestyle” rather than just its total production volume. *F. nucleatum* possesses unique adhesins (FadA) that allow it to adhere firmly to CRC cells and form biofilms directly on the tumor surface [7]. We hypothesize that this adhesion generates a concentrated “hotspot” of indole within the tumor microenvironment. In this scenario, even if the total amount of indole produced by *F. nucleatum* is small compared to *E. coli*, the local concentration directly exposed to the tumor cells could reach biologically active levels sufficient to activate AhR/c-Myc signaling—levels that free-floating bacteria cannot deliver to the epithelial surface due to dilution effects. While direct measurement of local indole gradients remains technically challenging, this concept aligns with established mucosal immunology principles, where localized high concentrations of microbial metabolites (e.g., short-chain fatty acids) specifically modulate host cell behavior at the crypt surface, distinct from their systemic effects. Validating this functional dichotomy—systemic load (driven by *E. coli*) vs. local concentration (driven by F. nucleatum)—represents a critical research opportunity to determine whether future therapies should prioritize systemic toxin reduction or microbiome modulation. Definitive verification will likely require advanced methodologies, such as in vivo stable isotope tracing or spatial metabolomics, to quantify local metabolite fluxes at the epithelial interface.

**Figure 1:**
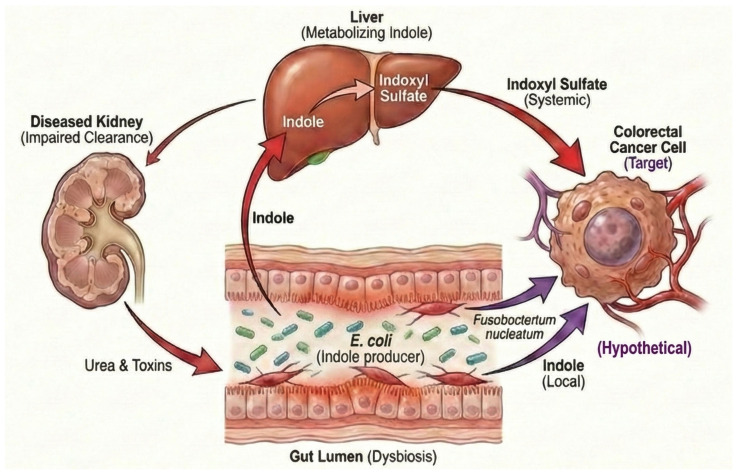
The Gut–Kidney Axis in Chronic Kidney disease (CKD)-Associated Colorectal Carcinogenesis. Molecular Mechanisms of Microbial Metabolite-Induced Colorectal Carcinogenesis. Signaling Pathways Activated by Systemic Indoxyl Sulfate and Local Indole in Colorectal Cancer (CRC) Cells. Uremic toxins and microbial metabolites activate two key pro-proliferative signaling pathways in CRC cells. (1) Systemic Indoxyl Sulfate and local indole (entering via a probenecid-sensitive transporter) act as Aryl Hydrocarbon Receptor (AhR) ligands, promoting *c-Myc* transcription (AhR pathway). (2) Systemic Indoxyl Sulfate activates the proto-oncogene Akt, leading to β-catenin stabilization and subsequent *c-Myc* upregulation (Akt pathway). Evidence suggests potential crosstalk, with Akt activation possibly enhancing AhR-mediated transcription. Both pathways contribute to Epidermal Growth Factor Receptor (EGFR) upregulation, increasing sensitivity to EGF and further promoting proliferation. Inhibitors targeting AhR (CH223191) and Akt (MK2206) can suppress these effects. (Note: Key experiments demonstrating these pathways, including EGFR upregulation, utilized the HCT-116 cell line, which harbors a *KRAS* mutation.) Anti-EGFR therapies are generally ineffective against *KRAS*-mutated tumors; thus, caution is required when interpreting potential therapeutic implications of EGFR targeting. The direct effect of local indole on *c-Myc* upregulation in this specific context is a hypothetical pathway proposed for future investigations.

The systemic accumulation of indoxyl sulfate represents a “double hit” phenomenon, characterized by increased production (systemic pool) and decreased renal clearance. Furthermore, indoxyl sulfate is over 90% protein-bound in plasma, which hinders its removal by hemodialysis [1]. This establishes a vicious cycle, as summarized in Figure 1.

### 3.3. The “Entry Mechanism Enigma” and Tumor Promotion

A critical question remains: How does indoxyl sulfate enter CRC cells? Although Ct-OATP1B3 is aberrantly expressed in CRC tissues [41,45], its primary localization to lysosomes suggests it functions in intracellular sequestration rather than surface uptake [41]. Furthermore, Enomoto et al. demonstrated that organic anion transporter 2 (OAT2) does not mediate indoxyl sulfate transport [46]. However, given that organic anion uptake is typically inhibited by probenecid, and considering reports such as Koto et al. showing that microcystin uptake is probenecid-sensitive [47], the existence of an unidentified ‘probenecid-sensitive organic anion transporter’ on the cell surface is strongly suggested. Identifying this specific transporter is a priority for future research to fully understand the uremic hijacking mechanism.

### 3.4. The Pro-Oncogenic Signaling Cascade: AhR, Akt, and Downstream Effectors

In vitro studies have shown that indoxyl sulfate, at concentrations comparable to those in patients with CKD (e.g., ≥62.5 µM), promotes CRC cell proliferation [48,49]. Indoxyl sulfate activates two key signaling pathways:

- The AhR/c-Myc Pathway: Indoxyl sulfate (and its precursor, indole) acts as an Aryl Hydrocarbon Receptor (AhR) ligand [7,48,49], leading to the upregulation of the oncogene *c-Myc* [7,49]. While indole is a known AhR ligand, it remains to be elucidated whether local indole accumulation in the tumor microenvironment directly induces *c-Myc* and cell proliferation in a manner similar to indoxyl sulfate. Investigating this potential direct action and the “crosstalk” between local indole and systemic indoxyl sulfate represents a critical research opportunity. Given their structural similarities and shared ability to act as AhR ligands, a direct effect is plausible, though differences in cellular uptake or metabolic conversion might modulate their relative potencies at the local level.
- Akt/β-Catenin/c-Myc Pathway: Indoxyl sulfate activates the proto-oncogene Akt, stabilizing β-catenin and increasing c-Myc expression [7,49].

The AhR Paradox: Context-Dependent Signaling. It is essential to recognize that AhR signaling is highly context-dependent, a phenomenon often described as the “AhR Paradox” [50,51,52,53,54]. In a healthy physiological state, AhR activation by dietary ligands (e.g., indoles from cruciferous vegetables) is crucial for maintaining intestinal barrier integrity, regulating intraepithelial lymphocytes, and ensuring immune tolerance [55,56,57,58,59]. However, the pathological outcome changes drastically in the context of CKD. We propose that under conditions of chronic uremic inflammation and systemic ligand overload (driven by indoxyl sulfate), AhR signaling skews towards a pathological, pro-oncogenic phenotype (e.g., immune suppression and *c-Myc* induction) rather than homeostatic maintenance [60,61,62,63,64]. This review focuses on this “dark side” of AhR specifically within the uremic milieu.

The existence of these converging pathways (both leading to c-Myc) suggests a robust proliferative drive in the cells. Inhibition by specific AhR antagonists (CH223191) and Akt inhibitors (MK2206) confirms their roles [48,49].

### 3.5. Amplifying Malignancy: Indoxyl Sulfate-Mediated Upregulation of EGFR

**Figure 2:**
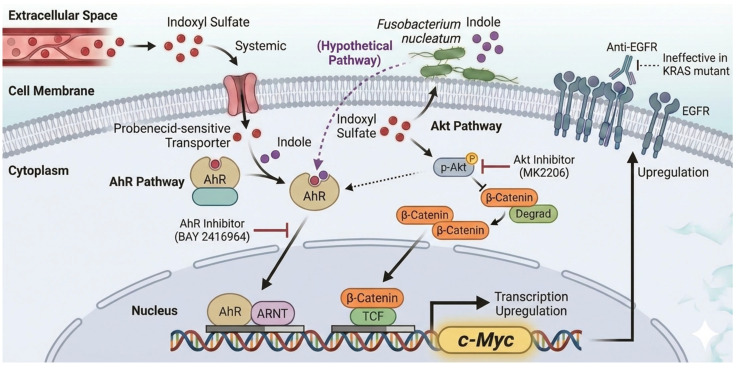
Molecular Convergence: AhR and Akt Pathways Driving Carcinogenesis. Schematic representation of the signaling pathways activated by uremic toxins and microbial metabolites in colorectal cancer cells. (1) Systemic Pathway (Solid Red Arrows): Systemic Indoxyl Sulfate enters the cell via an unidentified surface transporter (functionally characterized as a probenecid-sensitive organic anion transporter [47]). Inside the cell, Indoxyl Sulfate activates the AhR Pathway and the Akt Pathway, both converging to upregulate c-Myc expression. Ct-OATP1B3 is depicted on the lysosomal membrane, serving as a cancer-specific biomarker rather than an entry gate [41]. (2) Local Pathway (Dashed Purple Arrows): We propose that *F. nucleatum* adherent to the tumor surface creates a local high-concentration indole environment. Due to its lipophilicity, indole may passively diffuse across the cell membrane and directly activate cytoplasmic AhR (Hypothetical Pathway). (3) Feedback & Therapy: The upregulation of c-Myc leads to EGFR overexpression. In *KRAS*-mutated tumors (e.g., HCT-116) where anti-EGFR therapy is ineffective, targeting upstream drivers with AhR inhibitors (e.g., BAY 2416964) or Akt inhibitors (e.g., MK2206) represents a potential therapeutic strategy.

Indoxyl sulfate also upregulates Epidermal Growth Factor Receptor (EGFR) [48], mediated via both AhR and Akt pathways, increasing sensitivity to EGF [49]. This may contribute to a more aggressive phenotype in the uremic environment. We have previously demonstrated that indoxyl sulfate enhances angiotensin II signaling by upregulating *EGFR* expression in vascular smooth muscle cells, suggesting that this mechanism may be a broad, tissue-independent pathological feature [65]. However, caution is essential regarding the direct therapeutic translation of anti-EGFR agents in CRC. Key experiments establishing these pathways utilized the HCT-116 cell line, which harbors a *KRAS* mutation [48,49]. In clinical practice, anti-EGFR therapies are generally ineffective against *KRAS*-mutated tumors [66]. This limitation paradoxically highlights the value of the indoxyl sulfate axis: because downstream EGFR blockade is often futile in this population, targeting the upstream drivers, specifically the AhR and Akt pathways, may represent a superior therapeutic strategy. By intercepting the signal before it converges on potentially resistant downstream effectors, we may bypass the therapeutic deadlock often observed in *KRAS*-mutated CRCs. However, given that key mechanistic insights currently rely heavily on the HCT-116 cell line, future validation using diverse preclinical models, such as patient-derived organoids or in vivo models representing different molecular subtypes, is essential to generalize these findings. Specifically, findings primarily derived from *KRAS*-mutant/MSI models (like HCT-116) require validation across a broader range of CRC molecular subtypes (e.g., *BRAF*-mutant, MSS) and normal colonic epithelium to confirm the universal applicability of targeting these upstream pathways. These signaling pathways are illustrated in Figure 2 and summarized in Table 2.

### 3.6. Nuanced Hypothesis: The Potential for Subtype-Specific Effects on Right-Sided Colorectal Cancer

The key in vitro studies on indoxyl sulfate were conducted using HCT-116 cells [9,48,49], which are characterized by a *KRAS* mutation and microsatellite instability (MSI), hallmarks often associated with right-sided CRC. This leads to a nuanced hypothesis that the pro-proliferative effects of indoxyl sulfate may be more pronounced in right-sided CRC [48,49]. This is supported by the fundamental biological differences in metabolic capacity between the proximal and distal colon [67,68] and recent patient-level metabolomic data identifying significantly upregulated indoxyl sulfate in right-sided tumors [69].

## 4. The Diagnostic and Prognostic Framework in the CKD-CRC Cohort

This section addresses the significant clinical challenges in the diagnosis and monitoring of CRC in patients with CKD.

### 4.1. The Unreliability of Conventional Markers: The Confounding Effect of Renal Function on CEA

The utility of conventional protein-based tumor markers, such as carcinoembryonic antigen (CEA), is significantly compromised in patients with CKD. Serum CEA levels are known to be elevated in CKD patients without malignancy, and these levels rise as kidney function deteriorates [70,71]. This unreliability can result in diagnostic confusion.

### 4.2. The Prognostic Power of Renal Function and Sarcopenia Markers

Impaired renal function, evaluated by estimated Glomerular Filtration Rate (eGFR), has been established as an independent prognostic factor for “non-cancer death” in patients with CRC, reflecting the systemic burden of comorbidities [33]. The utility of Cystatin C alone as a CRC-specific prognostic marker remains to be fully elucidated. Therefore, the Cr/CysC ratio may serve as a dual indicator reflecting both renal filtration function and the systemic catabolic state. Crucially, this link is not merely observational; Sato et al. demonstrated that indoxyl sulfate directly accumulates in skeletal muscle, impairing mitochondrial function and inducing myoblast atrophy (uremic sarcopenia) [72]. Mechanistically, recent studies have further elucidated that this IS-induced skeletal muscle impairment is governed by an AhR-PDK4 axis; specifically, AhR activation upregulates Pyruvate Dehydrogenase Kinase 4 (PDK4), which in turn inhibits the pyruvate dehydrogenase complex, leading to mitochondrial dysfunction and energetic impairment [73]. This suggests that uremic toxins may act as a ‘metabolic accelerator’ for cancer cachexia. In CKD-CRC patients, the convergence of tumor-derived cytokines and uremic toxins creates a ‘Gut–Kidney–Muscle Axis’ of catabolism, making the management of IS levels critical not just for renal protection, but for preserving physical function and chemotherapy tolerance.

### 4.3. The Next Frontier in Diagnostics: Renal-Independent Blood-Based DNA Methylation Markers

The limitations of protein markers have spurred the search for alternative markers. Blood-based DNA methylation biomarkers, such as methylated Septin 9 (*SEPT9*) [74,75,76,77,78,79] and methylated Syndecan-2 (*SDC2*) [74,80,81], are promising. The key advantage of these markers in the CKD population is that they are DNA-based, not protein-based; therefore, their levels are not directly affected by renal clearance. However, it is important to note that large-scale validation studies specifically within the CKD population are still required to fully establish their diagnostic accuracy and utility.

### 4.4. Toward an Integrated Biomarker Panel for Personalized Risk Stratification

The future of diagnostics lies in the combination of different classes of biomarkers. A truly personalized management plan could be developed by creating an integrated conceptual panel that combines a CRC diagnostic marker (e.g., *SEPT9/SDC2*), a systemic vulnerability marker (e.g., CysC), and Gut–Kidney Axis markers. Indoxyl sulfate has vast potential as an “integrative biomarker” [7,34]. Clinically, this could be applied by measuring indoxyl sulfate levels in separate compartments. However, this conceptual integrated panel requires rigorous clinical validation to transition from a theoretical model to clinical utility. These studies will need to carefully consider potential confounding factors related to CKD, such as systemic inflammation or altered epigenetic profiles, that might influence methylation patterns beyond the presence of CRC.

- Urine Indoxyl Sulfate Dynamics: In early-stage CKD, elevated urinary excretion may reflect high gut production. However, in advanced CKD, urinary excretion naturally decreases due to impaired clearance, contributing to systemic accumulation. Thus, interpreting urine levels requires normalization for residual renal function (e.g., using the urine indoxyl sulfate-to-creatinine ratio). Furthermore, we propose a novel panel: while serum indoxyl sulfate reflects the total systemic load (a result of impaired clearance), urinary indoxyl sulfate (normalized to creatinine) may serve as a surrogate marker for ‘gut dysbiosis activity’ or intestinal production rate. Differentiating between ‘high producers’ and ‘poor excretors’ is crucial for selecting therapies.
- Elevated Serum Indoxyl Sulfate: Indicates systemic overload and impaired clearance.
- Fecal/tissue *F. nucleatum*: Quantifying abundance could serve as a proxy for local high indole production [7].
- Dual-Biomarker Strategy: Since colonoscopy and biopsy are standard of care for CRC diagnosis, assessing Ct-OATP1B3 expression levels in tumor tissues via immunohistochemistry adds minimal burden to the clinical workflow. By combining this tissue marker (serving as a highly specific ‘malignancy marker’ [41,45]) with serum indoxyl sulfate levels (representing the toxin ‘supply’), clinicians could identify a ‘super-high-risk’ subgroup—patients with both high systemic toxin load and high tumor susceptibility. Furthermore, this strategy may extend beyond the CKD population. Given that serum indoxyl sulfate levels are elevated not only in experimental models [82] but also in non-CKD CRC patients due to gut dysbiosis [69], Ct-OATP1B3 expression could serve as a universal predictive marker for responsiveness to microbiome-targeted therapies or oral adsorbents (AST-120), regardless of renal function (Table 3).

## 5. Therapeutic Strategies: From Toxin Reduction to Targeted Intervention

This section critically evaluates current and future therapeutic approaches, specifically repositioning the oral adsorbent AST-120 not merely as a renal protective agent, but as a potential disruptor of the oncogenic Gut–Kidney Axis.

### 5.1. The AST-120 Paradox: Analyzing Discrepant Results from the EPPIC Trials

The oral sorbent AST-120 (KREMEZIN^®^) acts by adsorbing indole precursors in the gut lumen, thereby reducing serum indoxyl sulfate levels [83]. Its efficacy in delaying CKD progression has been a subject of debate, particularly following the EPPIC-1 and EPPIC-2 trials, which showed no significant benefit in the overall analysis [84]. However, a crucial post hoc analysis of the US cohort revealed a significant delay in disease progression among the “high-compliance” subgroup (taking >80% of prescribed doses) [85]. This discrepancy offers a vital lesson for oncological applications: “Dose-Dependent Toxin Reduction.” If the therapeutic goal is to suppress the AhR/c-Myc carcinogenic axis, a partial reduction in indoxyl sulfate may be insufficient. Consistent adherence to reduce serum toxins below a specific “oncogenic threshold” is likely required to achieve biological prevention. This underscores the critical need for biomarker-enriched strategies (e.g., monitoring serum IS levels) in future trials to ensure adequate suppression of the targeted pathway (Table 4).

### 5.2. Beyond Renal Protection: AST-120 as a Potential “Upstream” Anti-Cancer Strategy

While clinical data on AST-120 specifically for CRC prevention is currently lacking, the mechanistic evidence reviewed in Section 3 provides a compelling rationale for its repositioning. We have demonstrated that systemic indoxyl sulfate drives CRC cell proliferation via the AhR/c-Myc and Akt pathways [48,49]. Therefore, by lowering the systemic pool of indoxyl sulfate, AST-120 theoretically acts as an “Upstream Signal Blocker,” preventing the ligand (IS) from reaching its receptor (AhR) on tumor cells. Unlike molecular targeted drugs (e.g., EGFR inhibitors) that block signals at the cell surface—and are often rendered ineffective by downstream mutations like KRAS—AST-120 targets the source of the ligand. This suggests that AST-120 could be effective regardless of the tumor’s genetic mutation status (KRAS wild-type or mutant), representing a universally applicable strategy for the CKD-CRC population.

### 5.3. Symptom Management and Quality of Life

Beyond survival, maintaining Quality of Life (QoL) is paramount in cancer care. AST-120 has demonstrated clear efficacy for uremic pruritus in a randomized trial, correlating with decreased serum indoxyl sulfate and TNF-α levels [86]. Since chronic inflammation and oxidative stress driven by uremic toxins also contribute to cancer cachexia and fatigue, reducing the toxin load may offer dual benefits: slowing tumor kinetics and alleviating systemic symptoms in palliative settings.

### 5.4. Microbiome and Dietary Modulation: A Strategy to Target the Source

Modulating the gut microbiota to reduce indole production is an alternative strategy. Approaches include the use of oral probiotics, such as *Bifidobacterium longum*, which has shown potential in reducing serum indoxyl sulfate levels [87], or synbiotics which have been investigated in trials such as SYNERGY [88,89]. Dietary modifications like low-protein diets to limit tryptophan availability for bacterial metabolism are also key [90]. Furthermore, dietary fiber intake and prebiotics (e.g., resistant starch) may modulate the gut environment [38,91]. For instance, certain fermentable fibers like inulin or resistant starch could promote beneficial bacteria that outcompete indole producers or alter gut pH to inhibit tryptophanase activity, although their optimal dosage and long-term safety in CKD need careful evaluation. However, therapeutic strategies must be nuanced. It is noteworthy that not all tryptophan metabolites are deleterious; for instance, a recent study demonstrated that indole-3-acetic acid (IAA) can ameliorate cachexia and reprogram systemic homeostasis [92]. Thus, the goal of microbiome intervention should not be the indiscriminate depletion of all indoles, but rather the restoration of a balanced metabolic profile (correcting dysbiosis) to favor beneficial metabolites over uremic toxins.

### 5.5. Novel Therapeutic Targets: Blocking the Downstream Effect

Indoxyl sulfate exerts proliferative effects via AhR and Akt pathways [7,9,49]. Crucially, a selective small-molecule AhR inhibitor, BAY 2416964, is currently in Phase I clinical trials for advanced solid tumors [93]. Akt inhibitors (e.g., Capivasertib, Ipatasertib, currently under investigation in solid tumors) also require further investigation. Given the prevalence of *KRAS* mutations in right-sided CRC and the consequent ineffectiveness of anti-EGFR therapies, targeting AhR and Akt represents a strategic bypass of this resistance mechanism, potentially offering a broader and more effective therapeutic window for the CKD-CRC population. However, given that AhR signaling also plays a physiological role in immune homeostasis [51] (e.g., regulating regulatory T-cell differentiation and cytokine production), prolonged systemic inhibition carries potential risks of immune dysregulation that must be carefully monitored. Additionally, since AhR signaling influences diverse physiological processes, including cardiovascular health and bone metabolism—systems already compromised in CKD—potential off-target effects in these organs warrant rigorous safety profiling.

### 5.6. A Note of Caution and Strategic Considerations for Targeted Therapies in CKD

Targeting the AhR and Akt pathways in the CKD population requires extreme caution due to the complex interplay between pharmacological vulnerability and physiological homeostasis. First, patients with CKD are characteristically frail, and their condition significantly alters drug pharmacokinetics and pharmacodynamics (PK/PD) due to changes in protein binding, metabolic enzyme activity, and renal clearance [94]. Given the high prevalence of polypharmacy in this population, rigorous assessments regarding potential drug–drug interactions and tolerability are essential before clinical application. Second, targeting AhR specifically presents a “double-edged sword.” Physiological AhR activation is essential for maintaining intestinal barrier integrity and orchestrating immune homeostasis, particularly through the regulation of innate lymphoid cells (ILC3) and regulatory T cells (Treg) [51]. Systemic and prolonged inhibition of AhR in CKD patients, who are already inherently immunocompromised, carries a theoretical but significant risk of exacerbating mucosal inflammation or increasing susceptibility to opportunistic infections. Therefore, the clinical translation of novel agents such as BAY 2416964 requires a sophisticated safety strategy. Future drug development should prioritize “gut-restricted” AhR modulators or nanoparticle-based delivery systems that specifically target tumor cells while sparing normal immune cells. This must be coupled with rigorous preclinical safety assessments in uremic animal models, followed by carefully designed Phase I trials specifically focused on safety and PK/PD parameters within the unique physiological environment of the CKD-CRC population.

### 5.7. Future Directions: A Combinatorial and Stratified Therapeutic Approach

**Figure 3:**
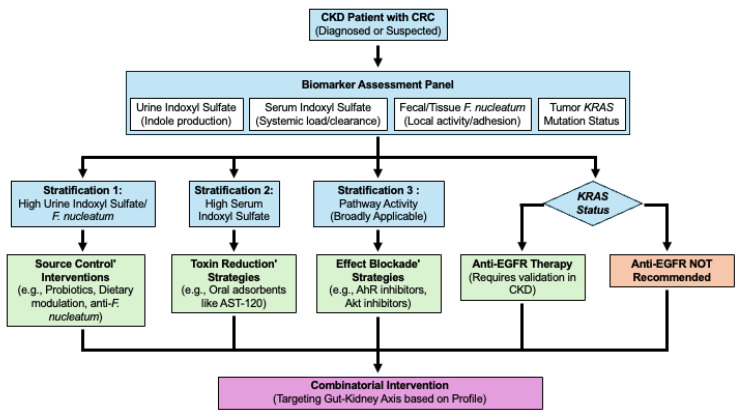
Conceptual Algorithm for Stratified Therapeutic Intervention in Chronic Kidney Disease (CKD)-Associated CRC: A Proposed Conceptual Framework for Personalized Treatment Strategies. This algorithm outlines a potential framework for personalizing therapeutic interventions in patients with CKD and colorectal cancer (CRC) based on the biomarker panel proposed in Section 4.4. Following CRC diagnosis or suspicion in a CKD patient, assessment includes measuring Urine Indoxyl Sulfate (indicator of indole production), Serum Indoxyl Sulfate (indicator of systemic load and impaired clearance), Fecal/Tissue *F. nucleatum* abundance (indicator of local activity/adhesion), and tumor *KRAS* mutation status. Patients could then be stratified as follows: (1) High Urine Indoxyl Sulfate AND/OR High Fecal/Tissue *F. nucleatum* Abundance, which might prioritize ‘Source Control’ interventions (for example, probiotics, dietary modulation, potentially anti-*F. nucleatum* strategies). (2) High Serum Indoxyl Sulfate levels may prioritize ‘Toxin Reduction’ strategies (e.g., oral adsorbents such as AST-120). (3) ‘Effect Blockade’ strategies (e.g., AhR inhibitors such as BAY 2416964 and Akt inhibitors) could be considered based on pathway activity and may be broadly applicable. Anti-EGFR therapy remains restricted to *KRAS* wild-type tumors, and its utility in this specific CKD context requires further validation. The algorithm emphasizes combining these approaches (Combinatorial Intervention) based on the individual’s biomarker profile to target the Gut–Kidney Axis comprehensively, as discussed in Section 5.6. (Note: This proposed algorithm is hypothetical and requires rigorous clinical validation before implementation).

Single-agent therapy is unlikely to be effective. The future lies in a stratified, combinatorial intervention strategy targeting the Gut–Kidney Axis at multiple points, potentially guided by an algorithm (Figure 3). It must be emphasized that this proposed algorithm represents a theoretical framework based on the “Gut–Kidney Axis” mechanisms discussed herein. It requires rigorous validation in prospective clinical trials to establish its safety and efficacy before clinical implementation.

- Source Control: High urine indoxyl sulfate/high fecal *F. nucleatum* → Probiotics, diet, anti-*F. nucleatum* strategies (e.g., targeted antibiotics or emerging “precision microbiome editing” approaches using bacteriophages). Notably, Wang et al. recently isolated novel bacteriophages capable of specifically lysing *F. nucleatum* without disrupting the beneficial commensal microbiota [95]. This represents a promising frontier for reducing the local indole load).
- Toxin Reduction: High Serum Indoxyl Sulfate → Oral sorbents (AST-120).
- Effect Blockade: Consider AhR antagonists (e.g., BAY 2416964) or Akt inhibitors, which are potentially less dependent on *KRAS*.

## 6. Conclusions and Strategic Recommendations for Clinical Practice and Future Research

### 6.1. Synthesis of Current Findings: A Causal and Mechanistically Driven Link

This review strongly supports a robust association and provides supporting evidence for a causal link between CKD and CRC, with clear pathophysiological mechanisms centered on gut microbial metabolite. We propose a “Gut–Kidney Axis” model in which CKD initiates gut dysbiosis, leading to increased systemic indoxyl sulfate production and local indole production. Impaired renal clearance causes systemic accumulation of uremic toxins. These metabolites promote CRC by activating AhR and Akt pathways.

### 6.2. Recommendations for Clinical Practice

- Enhanced Surveillance: Implement aggressive, personalized CRC screening for patients with CKD, especially high-risk subgroups (younger, diabetic women). This may involve considering earlier initiation of screening, shorter surveillance intervals, or utilizing the proposed non-invasive biomarker panel to triage high-risk individuals, given the potential challenges of conventional colonoscopy in this fragile population.
- Biomarker Interpretation: Use CEA cautiously. Advocating for validated, unconfounded biomarkers (*SEPT9/SDC2*) and research integrating urine/serum indoxyl sulfate and *F. nucleatum* detection.
- Perioperative management: A multidisciplinary approach should be employed to optimize renal and cardiovascular status preoperatively. Treatment decisions must always balance the oncological benefits against competing risks associated with CKD.

### 6.3. Imperatives for Future Research: Addressing Key Evidence Gaps

- Validation of Subgroup Risk: As noted in Section 2, the lack of granular data prevents precise risk estimation for younger patients and specific CKD stages. Future prospective cohorts are strictly necessary to define these absolute risk estimates and identify interaction effects between risk factors.
- Clinical Validation of Biomarkers: Validate DNA methylation markers and the indoxyl sulfate panel in large CKD cohorts.
- Stratified Therapeutic Trials: Future intervention trials (indoxyl sulfate-lowering) must use enrichment strategies (e.g., high baseline levels).
- Preclinical Validation of Targeted Therapies: Confirm the efficacy of AhR antagonists (e.g., BAY 2416964) and Akt inhibitors in CKD-CRC models.
- Investigation of the right-sided CRC hypothesis: Correlation of baseline indoxyl sulfate levels with tumor location and molecular subtype (*KRAS* and MSI).
- Quantitative Microbiome Contribution: Implement large-scale shotgun metagenomics to definitively quantify the relative contributions of *F. nucleatum* and *E. coli* to the indole pool, validating the “two-compartment” model.

### 6.4. Limitations and Future Directions

While this review synthesizes the current understanding of the Gut–Kidney Axis in CRC, several limitations in the existing evidence base must be acknowledged:

- Lack of Dose–Response Data: While in vitro studies demonstrate concentration-dependent effects of indoxyl sulfate [48], clinical evidence establishing a clear linear dose–response relationship between serum indoxyl sulfate concentrations and CRC incidence is currently lacking. Future studies should aim to define specific “risk thresholds” for serum toxins.
- Molecular Subtype Specificity and Patient-Derived Models: Current mechanistic insights, including the AhR/Akt/c-Myc axis, are predominantly derived from HCT-116 cell lines, which harbor *KRAS* mutations and MSI. It remains unclear whether this uremic toxicity is a universal driver across all molecular subtypes. To generalize these findings, urgent validation is required using diverse preclinical models, particularly Microsatellite Stable (MSS) lines (e.g., Caco-2, SW480) and Patient-Derived Organoids (PDOs) established from CKD patients. Such studies should aim to determine if the “uremic hit” is subtype-dependent, directly influencing patient stratification.
- Validation of the “Local Hotspot” Theory: While the “Two-Compartment Model” offers a logical explanation for the localized aggression of CRC in CKD, it is imperative to acknowledge that the existence of high-concentration indole “hotspots” remains a theoretical construct derived from mechanistic deduction. Direct quantification of the indole gradient at the bacterial–epithelial interface has not yet been achieved. Validation of this model represents a critical frontier; future studies utilizing advanced technologies such as spatial metabolomics (MALDI-MSI) or in vivo biofilm models are strictly necessary to definitively prove that local concentrations exceed systemic levels and are sufficient to drive AhR signaling in the tumor microenvironment.
- Potential for Novel Molecular Imaging: Based on the “Transporter-Gated” hypothesis, we propose a novel diagnostic application: Indoxyl Sulfate-based Molecular Imaging. Since Ct-OATP1B3 is aberrantly expressed in CRC tissues but virtually absent in normal colonic epithelium, radiolabeled or fluorescent analogues of indoxyl sulfate could serve as specific imaging probes. Unlike conventional FDG-PET, which relies on glucose metabolism and can show false positives in inflammatory lesions, an OATP-targeted probe would specifically visualize cells possessing the “toxin uptake machinery.” Developing such probes could enable the early detection of “high-risk” precursor lesions that are metabolically primed to accumulate uremic toxins, effectively bridging the gap between mechanistic understanding and preventive oncology.
- Elucidation of the Cellular Entry Mechanism: While Ct-OATP1B3 is a specific marker for CRC, its lysosomal localization suggests it does not mediate the initial uptake of indoxyl sulfate. Identifying the specific plasma membrane transporter responsible for indoxyl sulfate entry in CRC cells is a priority to fully understand the “Transporter-Gated” toxicity.

## 7. Materials and Methods

Generative AI (Gemini, Google) was utilized during the preparation of this review specifically to assist in conceptualizing the figure designs and the logical flow of the "two-compartment model" as illustrated in Figure 1 and Figure 2. The authors provided specific scientific prompts to the AI tool, and the resulting conceptualizations were critically reviewed, verified for scientific accuracy, and extensively refined by the authors to ensure scientific integrity. The authors remain fully responsible for the final content and conclusions of the manuscript.

## Figures and Tables

**Table 1:** Key Epidemiological Findings on the Association between CKD and CRC.

Study Type	Population Studied	Effect Measure Type	Value (95% CI)	Key Findings
Meta-analysis	Overall CKD Patients	Pooled SIR	1.33 (1.30–1.36)	Significant increase in relative CRC incidence
Meta-analysis	eGFR < 60 vs ≥60	IRR	1.35 (1.12–1.63)	Increased cancer risk with lower eGFR
Meta-analysis + MR Analysis	Overall CKD Patients	Pooled RR	1.332 (1.084–1.637)	Significant increase in relative CRC risk
Meta-analysis + MR Analysis	Overall CKD Patients	MR OR	1.171 (1.063–1.289)	Genetically predicted CKD associated with increased CRC risk
Meta-analysis + MR Analysis	CKD Patients < 50 yrs	Pooled RR	2.119 (1.205–3.725)	More pronounced association in younger individuals
Meta-analysis + MR Analysis	Female CKD Patients	Pooled RR	1.550 (1.121–2.144)	More pronounced association in women
Cohort Study	Female CKD+DM Patients	HR	2.00 (vs. Controls) *	Synergistic risk increase from CKD and DM
Cohort Study	CKD Patients < 50 yrs	HR	3.7 (1.83–7.49) *	Very high risk in younger individuals

SIR: Standardized Incidence Ratio; RR: Risk Ratio; OR: Odds Ratio; HR: Hazard Ratio; IRR: Incidence Rate Ratio. * HR compares risk relative to a specific control group defined in the study.

**Table 2:** Proposed Mechanisms of Microbial Metabolite-Mediated Colorectal Carcinogenesis.

Mediator	Source/ Location	Target Receptor/ Molecule	Signaling Pathway	Downstream Effect	Cellular Outcome
Indoxyl Sulfate (Systemic)	Systemic Circulation/Liver metabolism	Unidentified Transporter → AhR	AhR pathway	Upregulation of *c-Myc*	Increased Cell Proliferation
Indoxyl Sulfate (Systemic)	Systemic Circulation/Liver metabolism	Unidentified Transporter /Mechanism → Akt	Akt pathway (Akt/β-Catenin/c-Myc)	Upregulation of *β-Catenin* and *c-Myc*	Increased Cell Proliferation
Indole (Local) *	*F. nucleatum* in TME	AhR (Direct diffusion?)	AhR pathway	Potential upregulation of *c-Myc*	Potential promotion of proliferation
Indoxyl Sulfate (Systemic)	Systemic Circulation/Liver metabolism	Unidentified Transporter /Mechanism → AhR and Akt	AhR and Akt pathways → c-Myc	Upregulation of *EGFR*	Enhanced EGF Sensitivity

* Note: The direct effect of local indole accumulation on *c-Myc* upregulation and cell proliferation in the tumor microenvironment is a hypothesis proposed in this review, warranting further investigation.

**Table 3:** Clinical Utility of Selected Biomarkers in the CKD-CRC Cohort.

Marker	Type	Clinical Utility	Utility in CKD	Potential Measurement Timing
CEA	Protein	Prognosis, monitoring	Confounded by renal function (Potentially enhanced by Akt)	Post-diagnosis, during/after treatment
Cystatin C	Protein	Prognosis, vulnerability	Independently predicts non-cancer death	Pre-operative, peri-operative
*SEPT9*	DNA Methylation	Diagnosis, screening	Not confounded by renal function	Screening (2nd line), diagnosis
sTNFR1	Protein	CKD progression prediction	Promising for predicting CKD progression	Baseline, longitudinal monitoring
Urine Indoxyl Sulfate	Uremic Toxin	Integrative biomarker (Gut Production Activity)	Reflects gut dysbiosis activity. High levels (normalized to Cre) suggest “High Producer” phenotype.	Baseline, monitoring of diet/ probiotic therapy
Serum Indoxyl Sulfate	Uremic Toxin	Integrative biomarker (Systemic Load)	Reflects total systemic burden (Production + Retention). Risk stratification.	Baseline, pre-op, monitoring
*F. nucleatum*	Bacterial Abundance	Integrative biomarker (Local Activity)	Reflects local adhesion/indole potential	Diagnosis, fecal screening

**Table 4:** Key Findings from Clinical Trials of AST-120.

Study Name	Study Design	Population	Primary Endpoint	Key Findings
EPPIC-1 & EPPIC-2	RCT, double-blind, placebo	Mod-severe CKD (n = 2035, non-stratified)	Time to Dialysis/Transplant/Cr Doubling	No significant difference overall (HR 1.03 & 0.91), suggesting limitations of non-stratified design
US Subgroup Analysis	Post hoc analysis	US Mod-severe CKD (n = 583, compliance ≥67%)	Time to primary endpoint	Significant delay in AST-120 group (HR 0.74), suggesting potential benefit in selected subgroups
Uremic Pruritus Trial	RCT	Hemodialysis patients (n = 99)	Pruritus Severity (VAS score)	Significant reduction in VAS score in AST-120 group

## Data Availability

No new data were created or analyzed in this study.

## Supplementary Materials

The following supporting information can be downloaded at: https://www.mdpi.com/article/10.3390/toxins18020072/s1. Methodology for AI-Assisted Figure Generation.
