# Supplementary material for: Indoxyl Sulfate in the Gut–Kidney Axis: Pathophysiology and Clinical Significance in CKD-Associated Colorectal Cancer
Source: Toxins (Basel). 2026 Jan 30;18(2):72. doi: 10.3390/toxins18020072 (PMC12944506; doi:10.3390/toxins18020072)
Supplement: Supplementary file 1 [file toxins-18-00072-s001.zip › toxins-4087923-supplementary.pdf]

**Supplementary Materials: Methodology for AI-Assisted Figure Generation**

## 1. Overview

To ensure transparency, reproducibility, and adherence to the ethical guidelines regarding digital images, we provide the detailed methodologies, tools, and prompts used to generate the schematic illustrations (Figure 1 and Figure 2) presented in the main text of the manuscript titled "Indoxyl Sulfate in the Gut-Kidney Axis: Pathophysiology and Clinical Significance in CKD-Associated Colorectal Cancer".

Figures 1 and 2 were generated using Gemini (Google). The generation process was strictly guided by specific biological constraints and design briefs defined by the authors. All generated images underwent rigorous quality control by the authors to ensure scientific accuracy and anatomical correctness before inclusion in the manuscript. (Note: Figure 3 was manually created by the authors and is not included in this AI generation report.)

## 2. Generation Details for Figure 1

- **Figure Title:** The Gut-Kidney-Colon Axis in CKD-Associated Colorectal Carcinogenesis.
- **Generation Tool:** Gemini (powered by the Imagen model; Google).
- **Date of Generation:** January 5, 2026
- **Input Prompt:**"A high-quality medical illustration of the 'Gut-Kidney-Colon Axis' in chronic kidney disease. The image should feature a cyclical layout connecting three main organs: (1) A shriveled, diseased kidney on the left, indicating impaired clearance. (2) A cross-section of the gut lumen at the bottom, showing dysbiosis with free-floating bacteria (labeled 'E. coli' (Indole producer)) and specific bacteria (labeled 'Fusobacterium nucleatum') firmly adhering to the epithelial wall. (3) A liver at the top, metabolizing indole. (4) A colorectal cancer cell on the right as the target. Visual flow: Red arrows depict 'Indoxyl Sulfate' circulating from the liver to the cancer cell (systemic route). Purple arrows depict 'Indole' moving directly from the adhered Fusobacterium to the cancer cell (local route), explicitly labeled as '(Hypothetical)'. The style should be clean, professional scientific art, white background, detailed, 8k resolution, photorealistic diagram."

## 3. Generation Details for Figure 2

- **Figure Title:** Molecular Convergence: AhR and Akt Pathways Driving Carcinogenesis.
- **Generation Tool:** Gemini (powered by the Imagen model; Google).
- **Date of Generation:** January 20, 2026
- **Input Prompt:**"A detailed molecular signaling pathway diagram inside a colorectal cancer cell. The image shows the cell membrane at the top and the nucleus at the bottom. Key components: (1) Systemic Pathway: Red solid arrows show 'Indoxyl Sulfate' entering the cell via Probenecid-sensitive Transporter. (2) Local Pathway: Purple dashed arrows show 'Indole' entering via passive diffusion directly through the lipid bilayer (labeled 'Hypothetical Pathway' in bold purple text) targeting Cytoplasmic AhR. (3) Two main signaling pathways in the cytoplasm: the 'AhR pathway' and the 'Akt pathway', both

converging on the gene 'c-Myc' inside the nucleus. (4) Upregulated 'EGFR' receptors on the membrane resulting from c-Myc activation. (5) Inhibitor symbols indicating blockage points for 'AhR Inhibitors' and 'Akt Inhibitors'. The style is a flat schematic diagram for a scientific journal, clear labels, distinct colors for pathways (Red for systemic, Purple for local/hypothetical), vector-style graphics, white background."

**4. Statement of Image Integrity**

The authors confirm that the images generated by the AI tool (Figures 1 and 2) were reviewed for scientific fidelity. No excessive post-processing, specific area filtering, or manipulation that would alter the interpretation of the scientific data was performed, in accordance with the journal's guidelines on image integrity. The text labels (specifically the purple bold formatting of '(Hypothetical Pathway)' to align with the dashed pathway) and specific arrow styles (dashed lines) within the figures were manually refined, verified, and formatted by the authors to ensure legibility, terminological accuracy, and distinction between proven and hypothetical pathways.
